# The intragenic epistatic association of *ADD3* with biliary atresia in Southern Han Chinese population

**DOI:** 10.1042/BSR20171688

**Published:** 2018-06-12

**Authors:** Zhe Wang, Xiaoli Xie, Jinglu Zhao, Ming Fu, Yonglan Li, Wei Zhong, Huimin Xia, Yan Zhang, Rui-Zhong Zhang

**Affiliations:** Department of Pediatric Surgery, Guangzhou Institute of Pediatrics, Guangzhou Women and Children’s Medical Center, Guangzhou Medical University, Guangzhou, Guangdong

**Keywords:** association, ADD3, Biliary Atresia, intragenic epistatic

## Abstract

Biliary atresia (BA) is a multifactorial pathogenic disease with possible genetic components. As a member of membrane skeletal proteins in the liver and bile ducts, a haplotype composed by five single nucleotide polymorphisms (SNPs) on adducin 3 (*ADD3*) has been identified as associated with BA. However, limited study was designed to further elaborate the mutual relationship amongst those replicated SNPs to disease. We selected three susceptibility SNPs in *ADD3* and conducted a replication study using 510 BA cases and 1473 controls to evaluate the individual function of the SNPs and further stratified the potential roles with disease and its subclinical features. Two SNPs in *ADD3* were replicated as associated with BA (1.60E-04 ≤ *P*≤1.70E-04, 1.33 ≤ odds ratio (OR) ≤ 1.58 for rs17095355, 2.10E-04 ≤ *P*≤5.30E-04, 1.26 ≤ OR ≤ 1.57 for rs2501577). Though we failed to replicate the individual association of rs10509906 to disease, the intragenic epistatic effect between rs10509906 and rs2501577 was suggested as exhibiting susceptibility to BA, further cross-validated by multifactor dimensionality reduction (MDR) (*P*=0.068, OR = 1.37), which may explain extra hidden heritability of *ADD3* to BA. Furthermore, through subclinical stratification, we also observed the association of risk to disease mainly came from the female patients.

## Introduction

Biliary atresia (BA) is a multifactorial pathogenic disease exclusively seen in infants. It is characterized by exacerbating jaundice and acholic stools caused by progressive intra- and extrahepatic bile duct fibrosis. The incidence of the disease varies amongst populations, ranges from 1 in 5000 in Asians to 1 in 18000 in Caucasians [1–3]. The etiology of BA is still unclear, several hypotheses have been raised, including perinatal virus infection, environmental toxins, and genetic defects [4,5].

Growing evidence indicates that BA is caused by genetic pre-exposures and later an environmental secondary strike [6]. Previous genome-wide association studies (GWASs) had identified common genetic variant (rs17095355) in 10q24.2 associated with BA [7]. Subsequently, Cheng et al. [8] revealed five single nucleotide polymorphisms (SNPs) in the intergenic region between X-prolyl aminopeptidase P (*XPNPEP1*) and adducin 3 (*ADD3*) as associated with BA, including rs17095355, rs10509906, rs2501577, rs6584970, and rs708605. Further, they found the risk haplotype may modulate the *ADD3* expression level which varies in different status of the disease. However, whether the haplotype association acquired according to the linkage equilibrium or potential intragenic effect was not clear.

In the present study, we conducted a replication study of three SNPs in *ADD3* using 510 BA cases and 1473 controls. The association of two SNPs in *ADD3* was further replicated including rs17095355 and rs2501577. However, we failed to replicate the individual effect of SNP rs10509906 to disease. Haplotype analysis amongst the three included SNPs presented stronger association with disease than each individual SNP alone. We further identified intragenic epistasis between rs10509906 and rs2501577 which may elevate the risk of disease.

## Materials and methods

### Study subjects

Tissue samples were taken during surgical procedures from 510 sporadic BA patients recruited from 2000 to 2015. They were all claimed as unrelated Southern Han Chinese. The blood samples of 1473 geographically and ethnically matched controls were collected with no history of BA and related hepatobiliary disorders. The samples included in the current study were collected from Guangzhou Women and Children’s Medical Center. The study was approved by the institutional review board of the hospital. Written informed consents were provided by guardians of all patient subjects. All cases have undergone ultrasonography evaluation before surgical procedures. Diagnosis was made by intraoperation cholangiography and confirmed by pathology after surgery.

### SNP genotyping and quality control

According to the previous study by Cheng et al. [8], five SNPs in *ADD3* were reported as associated with BA, SNPs were annotated and scored according to RegulomDB (Supplementary Table S1).The detailed clinical information adopted for further analysis to the selected SNPs was summarized in Supplementary Table S2. Three SNPs (rs17095355, rs10509906, and rs2501577) in *ADD3* were included according to the relative higher annotation score and lower linkage disequilibrium (LD, *r^2^*< 0.8) in between as shown in Supplementary Figure S1. The samples were genotyped by MassARRAY iPLEX Gold system (Sequenom) based on the manufacturer’s instructions. The quality control steps were carried out as follows: all three SNPs passed the quality control with less than 10% missing data. SNPs were removed if Hardy–Weinberg equilibrium (HWE) *P*<0.05 calculated by control subjects.

**Table 1:** Replication results on three SNPs in *ADD3* in Southern Han Chinese population using 510 cases and 1473 controls

SNP	CHR	Gene	Left_gene	Right_gene	Cheng et al. (*Hepatology*, 2013) [8]	BP	A1/A2	Cases	Controls	OR (CI 0.95)	*P*	*P*_adj
				*P_hwe=0.55*								
**rs17095355**	10	*NA*	*XPNPEP1*	*ADD3*	*MAF case/control): 0.54/0.39*	109975992	T/C	461/483	1212/1680	1.33 (1.15 ∼1.54)	**1.60E-04**	**1.20E-04**
					*OR: 1.83 (1.45–2.31)*		ADD	-	-	1.34 (1.15∼1.56)	**1.50E-04**	**2.58E-04**
					*RISK allele: T*		DOM	358/114	964/482	1.58 (1.24∼2.00)	**1.70E-04**	**2.27E-04**
					*P-value: 5.32E-06*		REC	103/369	248/1198	1.35 (1.04∼1.75)	0.02	0.03
				*P_hwe=0.53*								
rs10509906	10	*NA*	*XPNPEP1*	*ADD3*	*MAF (case/control): 0.14/0.23*	109997916	C/G	200/786	610/2294	0.95 (0.79 ∼1.14)	0.58	0.53
					*OR: 0.52 (0.38–0.71)*		ADD	-	-	0.96 (0.80∼1.15)	0.64	0.75
					*RISK allele: G*		DOM	183/310	542/910	0.99 (0.80∼1.22)	0.92	1
					*P value: 4.91E-05*		REC	17/476	68/1384	0.75 (0.43∼1.28)	0.29	0.26
				*P_hwe=0.87*								
**rs2501577**	10	*ADD3*	*XPNPEP1*	*MXI1*	*MAF (case/control): 0.54/0.42	110086929	G/A	461/479	1237/1671	1.31 (1.13 ∼1.51)	**3.60E-04**	**4.03E-04**
					*OR: 1.61 (1.28–2.03)*		ADD	-	-	1.30 (1.12∼1.51)	**5.30E-04**	**4.29E-04**
					*RISK allele: G*		DOM	357/113	972/482	1.57 (1.24∼2.00)	**2.10E-04**	**2.17E-04**
					*P-value: 8.28E-05*		REC	104/366	265/1189	1.26 (0.98∼1.63)	0.07	0.06

A1/A2 indicate the risk allele and protective allele to disease. F_A/F_U indicates risk allele frequency of the SNP in cases or controls. The *P*-value indicates the significance based on allelic association tests. The calculation of OR is also based on the risk allele of each SNP. Abbreviations: BP, base pair where the SNP is located; CHR, chromosome; Gene.refgene, the gene where the SNP is located at; OR, odds ratio.

* Shows the association of rs2501575 which is in full LD (*r^2^* = 1) with rs2501577 in Chinese.

### Association analysis

SNP association were analyzed in a logistic model to detect the association between all SNPs and BA risk by comparing the allele frequency in patients and controls (basic allelic test) using PLINK1.9. Odds ratios (ORs) and corresponding 95% confidence intervals (CIs) were calculated from the logistic regression model. Genotype test of 3 × 2 contingency tables, Cochran–Armitage trend test, test of the additive model, dominant and recessive model tests were also performed by PLINK1.9. [9].

### Independence testing

HaploView was utilized to generate LD patterns and values in our study. Independent contribution tests for SNPs in *ADD3* toward disease associations were done using logistic regression adjusting for the effect of a specific SNP in the same locus (SNPTEST v2.5b). [10].

### Haplotype association analysis

First, all founders were phased using the E-M algorithm implanted in PLINK, which will generate haplotype-specific tests (1 df) for both disease and quantitative traits; an omnibus association statistic was also computed (P_omnibus). In all the cases, the tests were based on the expected number of haplotypes each individual had. Then the association was performed on all the most likely haplotype assignments as SNPs and use all the standard analytic options (*P*). The case/control omnibus test is a H-1 degree of freedom test, if there are H haplotypes.

### Genetic epistasis effect analysis

Epistasis test (case–control analysis) was performed using parametric logistic regression (PLINK1.9) [11]. To further clarify the epistasis effect, we also used multifactor dimensionality reduction (MDR) analysis [12] to perform the pairwise non-parametric epistasis test. This method includes a cross-validation (CV) procedure as well as a permutation test which minimizes false positive results by multiple examinations of the data. By comparing the average prediction error from the observed data with the distribution of average prediction errors under the null hypothesis, the statistical significance was determined. The MDR analysis was carried out using version 2.0 of the open-source MDR software package (http://www.epistasis.org).

## Results

### Association of *ADD3* SNPs with BA

Three previously identified SNPs in *ADD3* were selected for replication using 510 patients and 1473 controls from South Chinese population (see selection details in ‘Materials and methods’ section). All the SNPs did not violate the HWE in the control subjects, further association analysis was performed as shown in Table 1. The association of SNP rs17095355 was successfully replicated (OR = 1.33, *P*=1.6E-04), consistent with the previous GWAS [7,8]. We also replicated the association of SNP rs2501577, showing a comparable significance with SNP rs17095355 (OR = 1.31, *P*=3.6E-04); but failed to replicate the association of SNP rs10509906 (*P*=0.58). To better understand any potential genetic inherited pattern, we specified the association following additive, dominant, and recessive models. For two replicated SNPs in *ADD3*, larger effect was observed in both SNPs under the dominant models (OR = 1.58 for rs17095655 and OR = 1.57 for rs2501577, respectively).

The LD amongst the three SNPs based on Guangzhou samples were examined. The LD pattern was consistent with the 1000G data, showing limited LD of SNP rs10509906 with other two replicated SNPs (*r^2^*< 0.2). SNP rs17095355 and rs2501577 showed moderate LD with each other (*r^2^* = 0.72). According to the pairwise independence test (Table 2), we observed rs17095355 remains significance after adjusting for the effect of SNP rs2501577 (*P*=0.032); however, SNP rs2501577 was not significant conditioning on the effect of SNP rs17095355 (*P*=0.86), which may reflect the association mainly derived from SNP rs17095355.

**Table 2:** Independence test by adjusting for the effects of other SNPs in the *ADD3* region

SNP	SNPs whose effects were adjusted*		
	rs17095355	rs10509906	rs2501577
rs17095355	NA	***P*=5.3E-05**	***P*****=0.032**
		1.42 (1.20–1.68)	1.38 (1.03–1.85)
rs10509906	*P*=0.13	NA	*P*=0.13
	1.17 (0.95–1.44)		1.18 (0.95–1.46)
rs2501577	*P*=0.86	***P*=9.5E-05**	NA
	0.97 (0.73–1.30)	1.41 (1.19–1.68)	

* The data in each column represent the remaining effect of association (*P*-values) after adjusting for the effect of SNP(s) on the top row of each column.

### Haplotype analysis

Haplotype was estimated using the standard E-M algorithm and Chi-square test was performed. We tested the association of different combinations on the three SNPs including rs17095355, rs10509906, and rs2501577 to BA (Table 3). More than one haplotype were observed as risk combinations associated with BA, the omnibus association was adopted to calculate the overall association of haplotypes to disease. For the replicated two SNPs (rs17095355 and rs2501577), the risk allele combination T-G showed more significant association with disease than each individual SNPs alone (OR equal to 1.37 and P equal to 4.82E-05 for the haplotype compared with OR equal to 1.33 and P equal to 1.6E-04 for the most associated SNP rs17095355). Interestingly, we also observed SNP rs10509906(G) may play potential role with SLE. We observed T-G-G formed by the risk allele of the three SNPs was the strongest risk haplotype with a combined OR of 1.38 and *P*-value equal to 1.88E-05. Thus, SNP rs10509906 may play unknown role, such as intragenic epistasis, which may elevate the risk to disease.

**Table 3:** The association of haplotypes derived from three SNPs on *ADD3*

SNP combination	Haplotype	F_A	F_U	DF	*P*	OR (CI 0.95)
rs17095355|rs10509906	OMNIBUS	NA	NA	2	4.23E-04	-
Risk	TG	0.48	0.41	1	**2.23E-04**	**1.32 (1.15–1.53)**
Protective	CG	0.32	0.38	1	5.07E-04	0.76 (0.65–0.88)
rs10509906|rs2501577	OMNIBUS	NA	NA	2	3.97E-04	-
Risk	GG	0.49	0.43	1	**2.25E-04**	**1.32 (1.15–1.53)**
Protective	GA	0.30	0.36	1	4.80E-04	0.75 (0.65–0.88)
rs17095355|rs2501577	OMNIBUS	NA	NA	3	2.67E-04	-
Risk	TG	0.46	0.39	1	**4.82E-05**	**1.37 (1.18–1.58)**
Protective	CG	0.02	0.04	1	0.028	0.59 (0.38–0.93)
Protective	CA	0.49	0.54	1	3.25E-03	0.80 (0.69–0.92)
rs17095355|rs10509906|rs2501577	OMNIBUS	NA	NA	4	2.19E-04	-
Risk	TGG	0.47	0.39	1	**1.88E-05**	**1.38 (1.19–1.59)**
Protective	CGG	0.02	0.04	1	0.047	0.64 (0.41–0.99)
Protective	CGA	0.29	0.34	1	1.79E-03	0.78 (0.66–0.91)

F_A indicates risk allele frequency of the SNP in each subclinical group. F_U indicates protective allele frequency of the SNP in each subclinical group. Abbreviation: DF, degree of freedom.

### Intragenic epistasis in *ADD3* to BA

Based on the haplotype association results, we speculated potential genetic epistasis amongst the three SNPs in *ADD3* involved in the present study may affect the risk to BA. Pairwise epistasis test was performed using logistic regression implemented in PLINK. We observed marginal epistatic association for rs10509906 and rs2501577 with BA as shown in Table 4 (*P*=0.068, OR = 1.37). This piece of data was consistent with the findings in haplotype association that risk haplotype combination slightly improved the association with disease. However, due to sample size limitation and larger sample size requirement for epistasis validation, the results failed to reach the statistical significance (*P*<0.05). To conquer this shortcoming, we adopted the classic non-linear regression analysis method, MDR, to cross-validate the findings through logistic regression. Significant pairwise interaction between rs10509906 and rs2501577 was observed as shown in Table 4 (left bottom panel).

**Table 4:** Pairwise epistatic interacting results amongst three independent variants in *ADD3* done by logistic regression and MDR

SNP			ADD3		
			rs17095355	rs10509906	rs2501577
			Logistic regression		
*ADD3*	rs17095355	MDR	NA	*P*_int=0.21	*P*_int=0.54
				1.22 (0.89–1.67)	0.94 (0.75–1.16)
	rs10509906			NA	***P*****_int=0.068**
					**1.37 (0.98–1.91)**
	rs2501577			**CVC = 10/10**	NA
				***P*=0.0001**	
				**1.52 (1.23–1.88)**	

Cross‐validation consistency (CVC) reflects the number of times MDR analysis identified the same model as the data were divided into different segments.

### Clinical stratification of risk haplotype and replicated SNPs in *ADD3* with BA

The presence of cystic structure at the hilus was usually considered as cystic BA (CBA), which involved improved bile drainage and better native liver survival rate after surgery [13,14]. The existence of the subclinical symptoms might have underlying genetic background. Consistent with the previous epidemiological finding reported the presence of CBA in 5–10% of the patients [13,15], in our study there are 8.7% (44 out of 506 patients) patients diagnosed as CBA [14]. There was also a report showing slightly higher female predominance in BA with an incidence ratio equal to 1.4 compared with 1 [16,17]. We examined our BA samples, the female to male ratio was opposite to the previous study equal to 1 compared with 1.4. We further examined whether there exist unrevealed associations between the specific subclinical presentation and the major risk haplotypes. We examined the replicated SNPs in *ADD3* through subphenotype-control analysis including CBA compared with controls, non-CBA compared with controls and patients with different genders. As shown in Table 5, we failed to observe any tremendous differences between classified subclinical types. Interestingly, we found the risk of T-G-G to disease mainly came from the female patients (*P*=9.10E-04) other than male patients (*P*=0.59). Consistently, the association of individual replicated SNP to disease in female patients was more significant compared with males. This result may partially give us some clue on the mild difference genders to BA, of course, further functional validation was required for clearly understanding the mechanism of disease.

**Table 5:** The association results of SNPs in *ADD3* to different subclinical features

Subphenotype	Comparison	Major risk haplotype			Replicated SNPs					
		T-G-G			rs17095355			rs2501577		
		F_A	F_U	P	F_A	F_U	P	F_A	F_U	P
CBA/non-CBA	44 CBA compared with 1473 controls	0.45	0.39	0.23	0.46	0.42	0.41	0.48	0.43	0.34
	462 non-CBA compared withs 1473 controls	0.47	0.39	**2.41E-05**	0.49	0.42	**2.19E-04**	0.49	0.43	**6.33E-04**
Gender	214 females compared with 506 controls	0.48	0.38	**9.10E-04**	0.50	0.41	**2.78E-03**	0.52	0.42	**1.41E-03**
	292 males compared with 967 controls	0.48	0.46	0.59	0.48	0.42	0.017	0.47	0.43	0.064

*P*, the patient‐only linear regression test between subclinical groups including CBA/non-CBA patients, female and male patients; F_A/F_U indicates risk allele frequency of the SNP in patients with/without the clinical features.

## Discussion

BA is known as a multifactorial pathogenic disease with genetic components. The development of GWAS offers a different way of insight into the etiology of the disease. Garcia-Barcelo et al. [7] found several SNPs including rs17095355 on chromosome 10q24.2 strongly associated with BA. This SNP is in the intergenic area and flanked by the *XPNPEP1* and *ADD3* genes. *ADD3* was found widely expressed in the liver and biliary epithelial cells and played roles in remodeling of membrane cytoskeleton at points of cell–cell contact. XPNPEP1 is also found in liver epithelial cells which is important in bile acid excretion. Cheng et al. [8] from the same team fine-mapped the region in a larger case–control group and found the SNP rs17095355 achieved significance with a *P*-value of 10^−10^. A risk haplotype comprising five SNPs (rs17095355, rs10509906, rs2501577, rs6584970, and rs7086057) was identified strongly correlating with BA. Furthermore, the risk haplotype tagged by 17095355 was proved to be a *cis*-regulator of *ADD3*, presented the haplotype significantly down-regulated *ADD3* gene expression in BA liver. We further examined the functional role of three included SNPs according to the public available Genotype Tissue Expression (GTEx) project as genetic consequences of molecular characterization. As shown in the Supplementary Figures S2 and S4, we observed the two replicated SNPs serve as liver-specific expression quantitative trait loci (eQTL) which exhibited the risk alleles were associated with the abnormal expression of *ADD3* (*P*=2.8E-03 for rs17095355, *P*=3.4E-05 for rs2501577). SNP rs10509906 showed no evidence of correlation with Add3 expression in liver, which was consistent with the lack of evidence of individual association for the SNP to disease in current study (Supplementary Figure S3).

So far, the association studies of *ADD3* SNPs were investigated in the context of LD, the independent effects and intragenetic epistasis of the SNPs has not been insighted before. We replicated the result of haplotypes of *ADD3* with the risk of BA in Southern Han Chinese population. Consistent with previous studies, the association of rs17095355 and rs2501577 were successfully replicated, however, the association of 10509906 was not significant in our cohort.

On the other hand, by comparing the haplotypes with single individual SNP, we found that the risk allele rs17095355(T) and rs2501577(G) combination showed more significant association with disease than each SNP alone. Interestingly, the combination of SNP rs10509906(G) and rs2501577(G) exhibited a stronger effect size to disease by comparing with SNP rs2501577 alone. These results indicate that epistasis effect amongst SNPs may play an important role in the onset of BA. Intragenetic epistasis was observed between rs10509906 and rs2501577. More and more evidence supporting the theory of interlocus interaction strongly influencing the risk of complex trait [18]. These findings may indicate the existence of synergy effect amongst rs10509906 and its nearby SNPs.

CBA is a special subclinical type of BA. It was usually diagnosed prenatally. Besides the characteristical cystic lesion at the hilus, the patient with CBA has better jaundice clearance rate and longer native liver survival time. Whether these distinctions are caused by a genetic variation or environmental interactions remain largely unknown. In the present study, we replicated SNPs in *ADD3* through CBA-control analysis, but we failed to observe any tremendous difference between subclinical types. Further efforts should be taken in finding variants associated with CBA in a study with larger sample size before any conclusion could be drawn.

## Conclusion

Although the mechanism of SNPs associated with BA risk still remains largely undetermined, we successfully replicated two common variants as associated with BA, our study proposed a possibility that epistasis effect may play roles in BA pathogenesis. Further investigation could focus on verification of such effect on transcription and gene expression level.

## Supporting information

**supplementary Figure 1:** The LD (r2) patterns of five SNPs in *ADD3* in Chinese from Beijing and Southern (CHB_CHS) and Utah residents with ancestry from northern and western Europe (CEU). The LD patterns of rs17095355, rs10509906, rs2501577, rs6584970 and rs7086057 between (CHB_CHS) and CEU populations are similar, which are slightly different with the LD pattern of rs17095355 in CHB_CHS populations, reflecting the potential population difference.

**supplementary Figure 2:** SNP rs17095355 played as an expression quantitative locus (eQTL) with *ADD3* across different tissues in human, especially in Central nervous system using GTEX tool (https://www.gtexportal.org/home/)

**supplementary Figure 3:** SNP rs10509906 played as an expression quantitative locus (eQTL) with *ADD3* across different tissues in human, especially in Central nervous system using GTEX tool (https://www.gtexportal.org/home/).

**supplementary Figure 4:** SNP rs2501577 played as an expression quantitative locus (eQTL) with *ADD3* across different tissues in human, especially in Central nervous system using GTEX tool (https://www.gtexportal.org/home/).

**Supplementary Table 1:** Annotation of five SNP of ADD3 identified by Cheng et al using Regulome DB.

**Supplementary Table 2:** Detailed clinical information of the subjects in this study

## Abbreviations

ADD3: adducin 3
BA: biliary atresia
CBA: cystic BA
GWAS: genome-wide association study
HWE: Hardy–Weinberg equilibrium
LD: linkage disequilibrium
MDR: multifactor dimensionality reduction
OR: odds ratio
SNP: single nucleotide polymorphism
XPNPEP1: X-prolyl aminopeptidase P

## References

[1] Shim W.K., Kasai M. and Spence M.A. (1974) Racial influence on the incidence of biliary atresia. Prog. Pediatr. Surg. 6, 53 4824812

[2] Petersen C., Harder D., Abola Z., Alberti D., Becker T., Chardot C. (2008) European biliary atresia registries: summary of a symposium. Eur. J. Pediatric Surg. 18, 111 10.1055/s-2008-1038479 18437656

[3] Hsiao C.H., Chang M.H., Chen H.L., Lee H.C., Wu T.C., Lin C.C. (2008) Universal screening for biliary atresia using an infant stool color card in Taiwan. Hepatology 47, 1233–1240 10.1002/hep.22182 18306391

[4] Balistreri W.F., Grand R., Hoofnagle J.H., Suchy F.J., Ryckman F.C., Perlmutter D.H. (1996) Biliary atresia: current concepts and research directions. Summary of a symposium. Hepatology 23, 1682 10.1002/hep.510230652 8675193

[5] Bezerra JA. (2006) The next challenge in pediatric cholestasis: deciphering the pathogenesis of biliary atresia. J. Pediatr. Gastroenterol. Nutr. 43 (Suppl. 1), S23–S29 10.1097/01.mpg.0000228197.28056.2f 16819397

[6] Hartley J.L., Davenport M. and Kelly D.A. (2009) Biliary atresia. Lancet 374, 1704 10.1016/S0140-6736(09)60946-6 19914515

[7] Garcia-Barcelo M.M., Yeung M.Y., Miao X.P., Tang C.S., Cheng G., So M.T. (2011) Genome-wide association study identifies a susceptibility locus for biliary atresia on 10q24.2. Hum. Mol. Genet. 19, 2917 10.1093/hmg/ddq196 PMC2893814 20460270

[8] Cheng G., Tang C.S., Wong E.H., Cheng W.W., So M.-T., Miao X. (2013) Common genetic variants regulating ADD3 gene expression alter biliary atresia risk. J. Hepatol. 59, 1285 10.1016/j.jhep.2013.07.021 23872602

[9] Purcell S., Neale B., Todd-Brown K., Thomas L., Ferreira M.A.R., Bender D. (2007) PLINK: a tool set for whole-genome association and population-based linkage analyses. Am. J. Hum. Genet. 81, 559–575 10.1086/519795 17701901 PMC1950838

[10] Marchini J., Howie B., Myers S., Mcvean G. and Donnelly P. (2007) A new multipoint method for genome-wide association studies by imputation of genotypes. Nat. Genet. 39, 906–913 10.1038/ng2088 17572673

[11] Purcell S., Neale B., Toddbrown K., Thomas L., Ferreira M.A., Bender D. (2007) PLINK: a tool set for whole-genome association and population-based linkage analyses. Am. J. Hum. Genet. 81, 559–575 10.1086/519795 17701901 PMC1950838

[12] Hahn L.W., Ritchie M.D. and Moore J.H. (2003) Multifactor dimensionality reduction software for detecting gene-gene and gene-environment interactions. Bioinformatics 19, 376–382 10.1093/bioinformatics/btf869 12584123

[13] Caponcelli E., Knisely A.S. and Davenport M. (2008) Cystic biliary atresia: an etiologic and prognostic subgroup. J. Pediatr. Surg. 43, 1619–1624 10.1016/j.jpedsurg.2007.12.058 18778995

[14] Komuro H., Makino S.I., Momoya T. and Nishi A. (2000) Biliary atresia with extrahepatic biliary cysts–cholangiographic patterns influencing the prognosis. J. Pediatr. Surg. 35, 1771–1774 10.1053/jpsu.2000.19248 11101734

[15] Arora A., Patidar Y., Khanna R., Alam S., Rastogi A. and Negi S.S. (2012) Cystic biliary atresia: confounding and intriguing. J. Pediatr. 161, 562 10.1016/j.jpeds.2012.04.066 22683035

[16] Wada H., Muraji T., Yokoi A., Okamoto T., Sato S., Takamizawa S. (2007) Insignificant seasonal and geographical variation in incidence of biliary atresia in Japan: a regional survey of over 20 years. J. Pediatr. Surg. 42, 2090–2092 10.1016/j.jpedsurg.2007.08.035 18082714

[17] Hopkins P.C., Yazigi N. and Nylund C.M. (2017) Incidence of biliary atresia and timing of hepatoportoenterostomy in the United States. J. Pediatr. 187, 253 10.1016/j.jpeds.2017.05.006 28746031

[18] Wei W.H., Hemani G. and Haley C.S. (2014) Detecting epistasis in human complex traits. Nat. Rev. Genet. 15, 722–733 10.1038/nrg3747 25200660

