# Supplementary material for: The intragenic epistatic association of ADD3 with biliary atresia in Southern Han Chinese population
Source: Biosci Rep. 2018 Jun 12;38(3):BSR20171688. doi: 10.1042/BSR20171688 (PMC5997795; doi:10.1042/BSR20171688)
Supplement: Supplementary file 1 [file bsr20171688_Supp1.pdf]

Supplementary Figure 1. The LD (r2) patterns of five SNPs in *ADD3* in Chinese from Beijing and Southern (CHB_CHS) and Utah residents with ancestry from northern and western Europe (CEU). The LD patterns of rs17095355, rs10509906, rs2501577, rs6584970 and rs7086057 between (CHB_CHS) and CEU populations are similar, which are slightly different with the LD pattern of rs17095355 in CHB_CHS populations, reflecting the potential population difference.

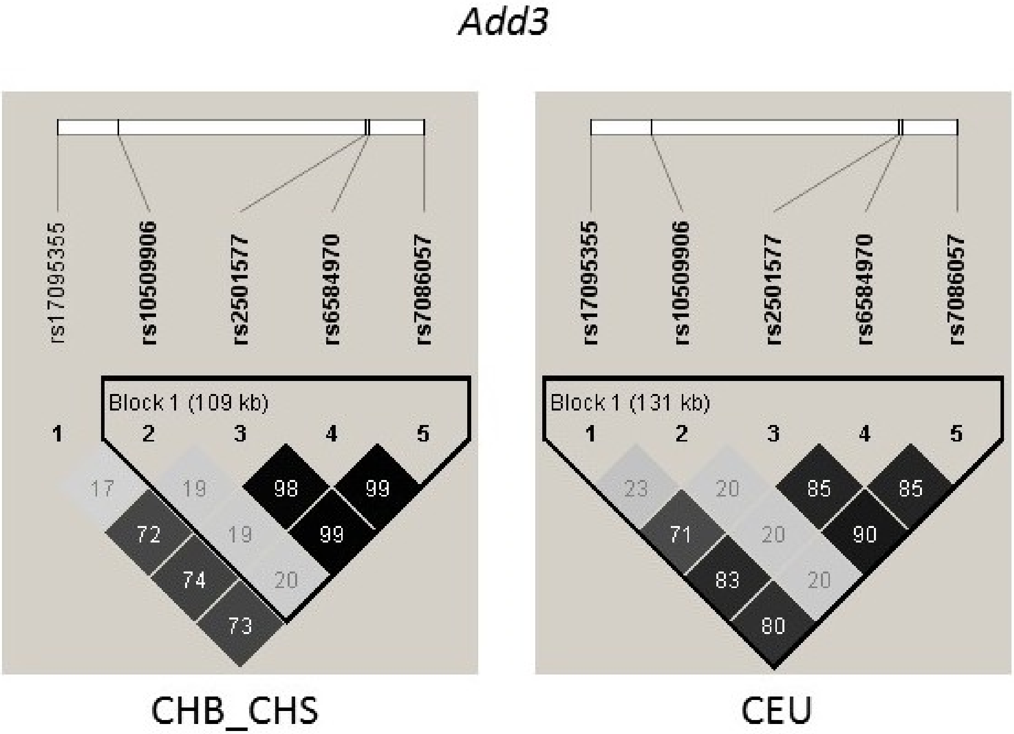

Supplementary Figure 2.SNP rs17095355 played as an expression quantitative locus (eQTL) with *ADD3* across different tissues in human, especially in Central nervous system using GTEX tool (https://www.gtexportal.org/home/)

Tissue	Samples	Beta	p-value	post-prob
Brain - Cerebellum	154	0.835	8.3e-24	1.00
Brain - Cerebellar Hemisphere	125	0.793	3.6e-18	1.00
Pituitary	157	0.589	4.1e-12	1.00
Uterus	101	0.530	4.1e-5	1.00
Spleen	146	0.433	7.3e-9	1.00
Ovary	122	0.370	0.01	0.960
Vagina	106	0.337	1.9e-3	0.982
Liver	153	0.279	2.8e-3	0.985
Artery - Aorta	267	0.273	1.3e-6	1.00
Stomach	237	0.257	2.3e-4	1.00
Colon - Transverse	246	0.254	1.0e-6	1.00
Small Intestine - Terminal Ileum	122	0.236	0.03	0.954
Prostate	132	0.217	0.008	0.940
Lung	383	0.189	1.5e-8	1.00
Colon - Sigmoid	203	0.181	1.1e-5	1.00
Adrenal Gland	175	0.178	0.02	0.989
Heart - Left Ventricle	272	0.177	5.1e-5	1.00
Nerve - Tibial	361	0.168	1.1e-7	1.00
Esophagus - Muscularis	335	0.157	5.1e-6	1.00
Cells - EBV-transformed lymphocytes	117	0.157	0.1	0.805
Artery - Tibial	388	0.142	7.8e-6	1.00
Adipose - Visceral (Omentum)	313	0.137	6.7e-5	1.00
Breast - Mammary Tissue	251	0.135	0.005	0.998
Brain - Frontal Cortex (BA9)	118	0.130	1.5e-3	0.991
Esophagus - Gastroesophageal Junction	213	0.123	0.005	0.968
Testis	225	0.117	0.009	0.949
Brain - Nucleus accumbens (basal ganglia)	130	0.114	0.03	0.935
Heart - Atrial Appendage	264	0.113	0.02	0.950
Thyroid	399	0.101	0.02	0.932
Muscle - Skeletal	491	0.0970	3.3e-3	0.943
Whole Blood	369	0.0945	1.8e-5	0.939
Brain - Putamen (basal ganglia)	111	0.0911	0.08	0.813
Brain - Anterior cingulate cortex (BA24)	109	0.0710	0.2	0.432
Adipose - Subcutaneous	385	0.0708	0.05	0.234
Brain - Hippocampus	111	0.0575	0.4	0.351
Skin - Not Sun Exposed (Suprapubic)	335	0.0571	0.2	0.327
Cells - Transformed fibroblasts	300	0.0509	0.1	0.00400
Minor Salivary Gland	85	0.0502	0.7	0.585
Brain - Caudate (basal ganglia)	144	0.0488	0.4	0.266
Brain - Spinal cord (cervical c-1)	83	0.0419	0.7	0.695
Brain - Hypothalamus	108	0.0358	0.7	0.425
Esophagus - Mucosa	358	0.0210	0.6	0.00
Brain - Cortex	136	0.0184	0.7	0.00
Skin - Sun Exposed (Lower leg)	414	0.0150	0.7	0.00
Artery - Coronary	152	0.00637	0.9	0.00800
Brain - Substantia nigra	80	-0.00736	0.9	0.327
Brain - Amygdala	88	-0.0133	0.9	0.223
Pancreas	220	-0.0407	0.5	0.00

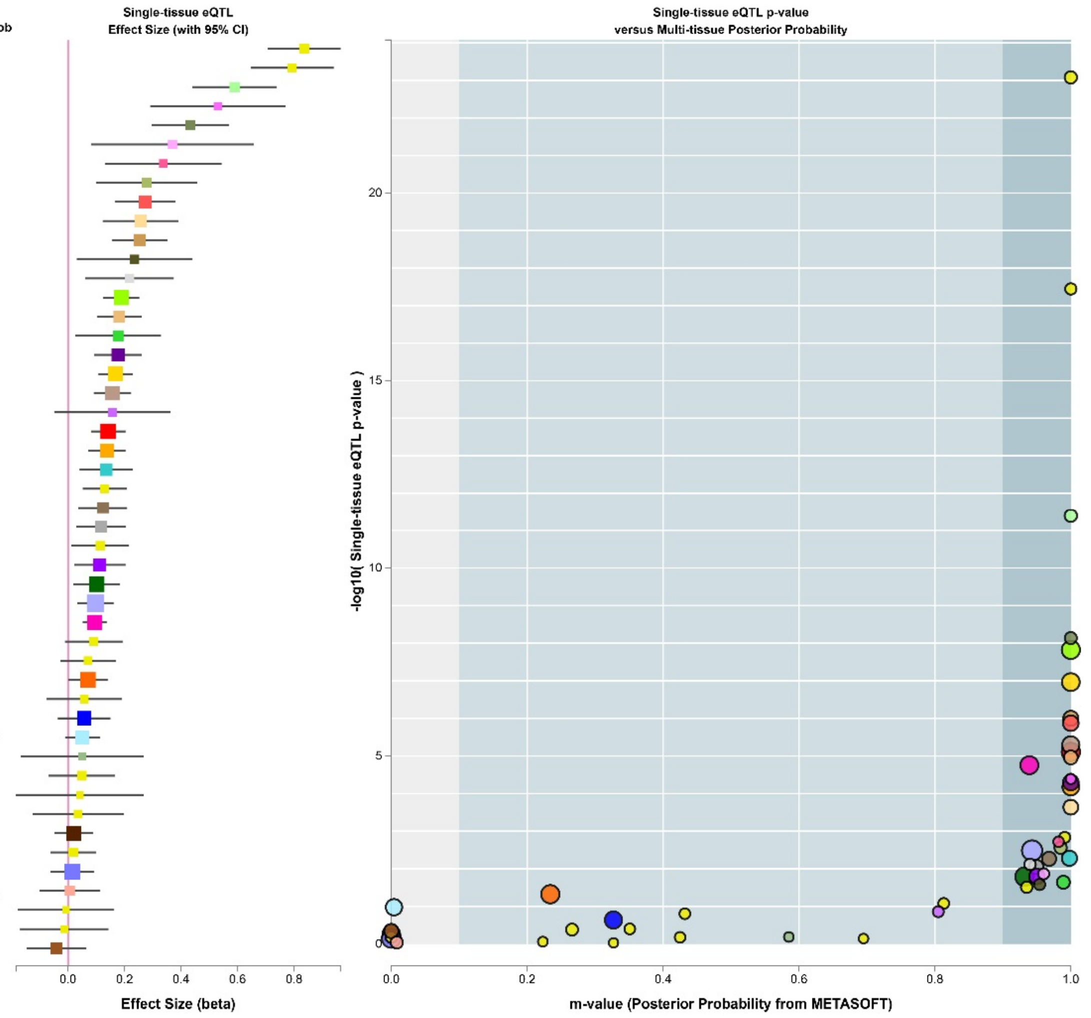

Supplementary Figure 3. SNP rs10509906 played as an expression quantitative locus (eQTL) with *ADD3* across different tissues in human, especially in Central nervous system using GTEX tool (https://www.gtexportal.org/home/).

Tissue	Samples	Beta	p-value	post-prob
Vagina	106	0.167	0.04	0.00
Brain - Spinal cord (cervical c-1)	83	0.0866	0.5	0.0950
Esophagus - Muscularis	335	0.0644	0.2	0.00
Colon - Sigmoid	203	0.0404	0.6	0.0370
Breast - Mammary Tissue	251	0.0304	0.5	0.00
Liver	153	0.0155	0.7	0.00
Colon - Transverse	246	0.0141	0.7	0.00
Brain - Nucleus accumbens (basal ganglia)	130	0.00824	0.9	0.00900
Brain - Hippocampus	111	0.00761	0.9	0.0500
Adipose - Subcutaneous	385	-0.00394	0.9	0.00
Small Intestine - Terminal Ileum	122	-0.0157	0.7	0.0140
Minor Salivary Gland	85	-0.0163	0.9	0.268
Testis	225	-0.0183	0.5	0.00
Heart - Left Ventricle	272	-0.0267	0.5	0.00
Adipose - Visceral (Omentum)	313	-0.0355	0.3	0.0120
Cells - Transformed fibroblasts	300	-0.0356	0.4	0.0310
Pancreas	220	-0.0387	0.4	0.0430
Thyroid	399	-0.0417	0.1	0.00
Muscle - Skeletal	491	-0.0468	0.06	0.00
Prostate	132	-0.0482	0.5	0.212
Esophagus - Mucosa	358	-0.0550	0.1	0.0370
Heart - Atrial Appendage	264	-0.0705	0.1	0.200
Artery - Tibial	388	-0.0708	0.03	0.122
Brain - Cerebellar Hemisphere	125	-0.0771	0.3	0.398
Artery - Aorta	267	-0.0817	0.07	0.321
Stomach	237	-0.0864	0.04	0.446
Brain - Cerebellum	154	-0.0915	0.2	0.482
Artery - Coronary	152	-0.0928	0.1	0.454
Brain - Amygdala	88	-0.0944	0.4	0.530
Brain - Substantia nigra	80	-0.101	0.4	0.450
Nerve - Tibial	361	-0.107	3.4e-3	0.888
Brain - Anterior cingulate cortex (BA24)	109	-0.114	0.4	0.547
Brain - Caudate (basal ganglia)	144	-0.121	0.09	0.710
Esophagus - Gastroesophageal Junction	213	-0.124	0.09	0.752
Brain - Frontal Cortex (BA9)	118	-0.126	0.2	0.773
Brain - Hypothalamus	108	-0.152	0.1	0.612
Whole Blood	369	-0.178	7.1e-8	1.00
Skin - Sun Exposed (Lower leg)	414	-0.187	3.7e-7	1.00
Uterus	101	-0.189	0.06	0.882
Lung	383	-0.192	4.9e-8	1.00
Brain - Putamen (basal ganglia)	111	-0.219	9.9e-4	0.989
Adrenal Gland	175	-0.221	4.3e-4	1.00
Brain - Cortex	136	-0.231	0.02	0.900
Skin - Not Sun Exposed (Suprapubic)	335	-0.232	2.7e-8	1.00
Pituitary	157	-0.242	3.6e-5	1.00
Ovary	122	-0.252	0.009	0.914
Spleen	146	-0.274	5.8e-4	0.988
Cells - EBV-transformed lymphocytes	117	-0.278	0.009	0.952

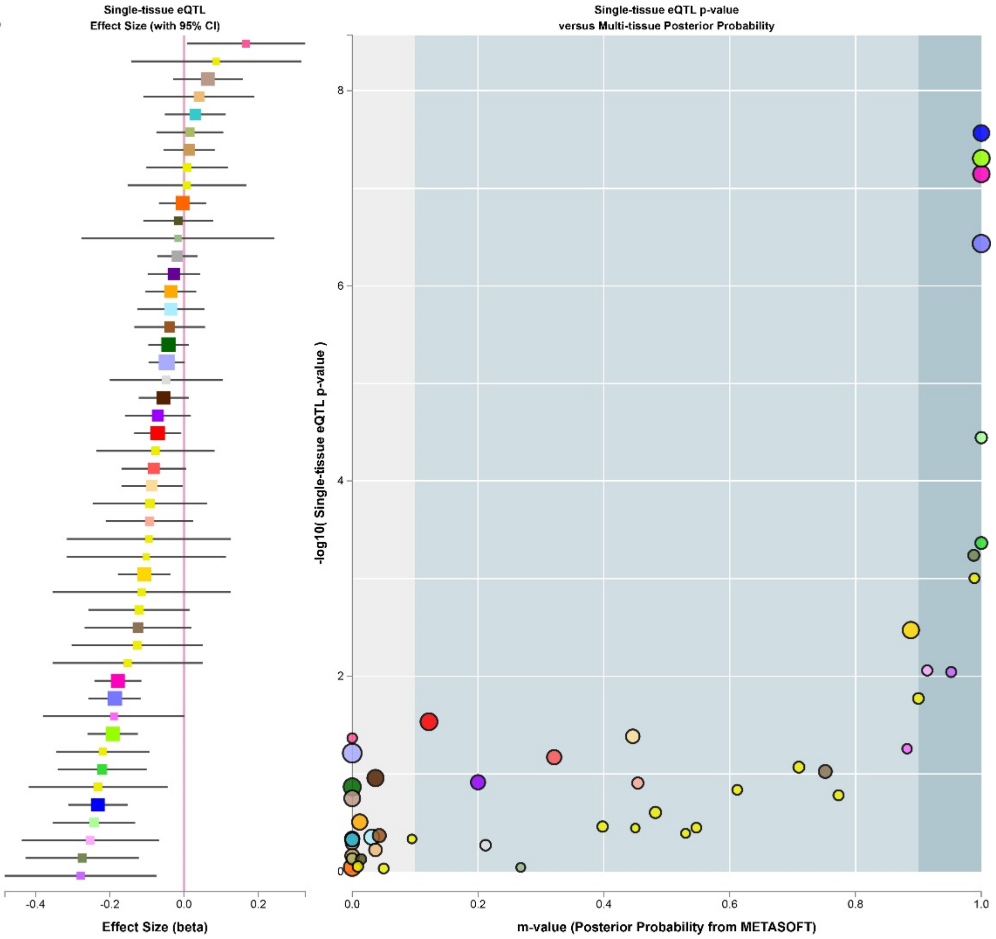

Supplementary Figure 4. SNP rs2501577 played as an expression quantitative locus (eQTL) with *ADD3* across different tissues in human, especially in Central nervous system using GTEX tool (https://www.gtexportal.org/home/).

Tissue	Samples	Beta	p-value	post-prob
Brain - Cerebellum	154	0.884	2.6e-23	1.00
Brain - Cerebellar Hemisphere	125	0.702	2.8e-12	1.00
Uterus	101	0.598	4.5e-5	1.00
Ovary	122	0.579	6.3e-4	0.998
Pituitary	157	0.565	2.0e-11	1.00
Spleen	146	0.540	1.3e-13	1.00
Artery - Aorta	267	0.425	4.2e-14	1.00
Liver	153	0.375	3.4e-5	1.00
Adrenal Gland	175	0.359	2.1e-6	1.00
Stomach	237	0.322	2.6e-6	1.00
Small Intestine - Terminal Ileum	122	0.303	1.4e-3	1.00
Colon - Transverse	246	0.285	1.9e-8	1.00
Colon - Sigmoid	203	0.254	2.3e-11	1.00
Nerve - Tibial	361	0.241	2.3e-15	1.00
Heart - Left Ventricle	272	0.209	2.0e-6	1.00
Thyroid	399	0.206	1.1e-6	1.00
Vagina	106	0.200	0.1	0.960
Breast - Mammary Tissue	251	0.196	4.7e-5	1.00
Lung	383	0.193	3.7e-9	1.00
Artery - Tibial	388	0.193	5.8e-10	1.00
Heart - Atrial Appendage	264	0.186	4.8e-5	1.00
Artery - Coronary	152	0.182	1.9e-3	1.00
Esophagus - Muscularis	335	0.180	8.3e-8	1.00
Adipose - Visceral (Omentum)	313	0.175	3.9e-7	1.00
Testis	225	0.170	1.2e-4	1.00
Brain - Putamen (basal ganglia)	111	0.154	2.9e-3	0.961
Muscle - Skeletal	491	0.149	2.8e-6	1.00
Esophagus - Gastroesophageal Junction	213	0.140	1.1e-3	1.00
Brain - Frontal Cortex (BA9)	118	0.139	1.1e-3	1.00
Whole Blood	369	0.137	8.3e-10	1.00
Skin - Not Sun Exposed (Suprapubic)	335	0.136	2.9e-3	0.988
Cells - EBV-transformed lymphocytes	117	0.128	0.2	0.851
Brain - Nucleus accumbens (basal ganglia)	130	0.120	0.03	0.910
Adipose - Subcutaneous	385	0.119	5.1e-4	0.988
Prostate	132	0.0997	0.2	0.776
Minor Salivary Gland	85	0.0971	0.4	0.790
Cells - Transformed fibroblasts	300	0.0957	4.1e-3	0.434
Pancreas	220	0.0872	0.09	0.445
Brain - Caudate (basal ganglia)	144	0.0860	0.1	0.621
Skin - Sun Exposed (Lower leg)	414	0.0788	0.04	0.197
Brain - Anterior cingulate cortex (BA24)	109	0.0674	0.2	0.195
Brain - Cortex	136	0.0456	0.2	0.00
Brain - Hippocampus	111	0.0456	0.5	0.279
Esophagus - Mucosa	358	0.0256	0.5	0.00
Brain - Substantia nigra	80	-0.0135	0.9	0.0760
Brain - Hypothalamus	108	-0.0263	0.7	0.0680
Brain - Spinal cord (cervical c-1)	83	-0.0430	0.7	0.170
Brain - Amygdala	88	-0.0656	0.4	0.0120

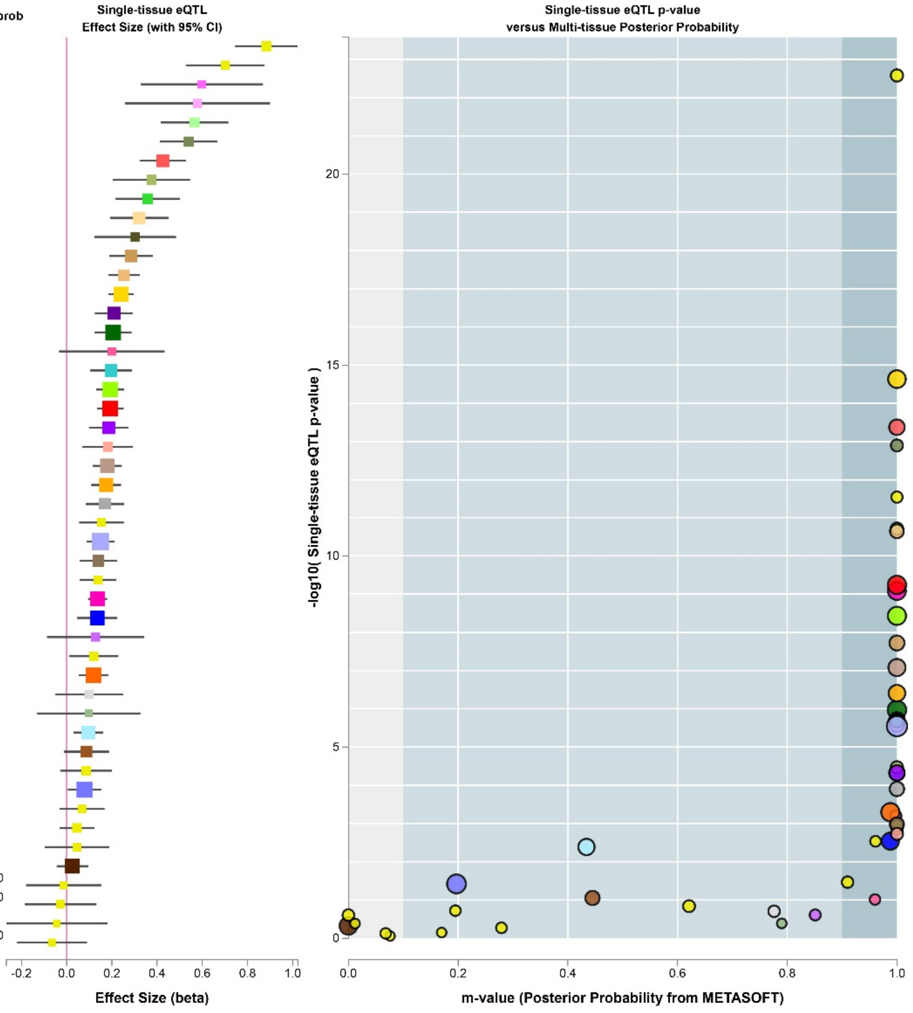

Supplementary Table 1. Annotation of five SNP of ADD3 identified by Cheng et al using Regulome DB.

#chromosome	rsid	hits	score*
chr10	rs17095355	Chromatin_Structure||DNase-seq|Hrpe, Chromatin_Structure||DNase-seq|Werirb1, Chromatin_Structure||DNase-seq|H7es	5
chr10	rs2501577	Motifs|PWM||RUNX3, Chromatin_Structure||DNase-seq|Tregwb83319432	5
chr10	rs6584970	Motifs|PWM||RFX1, Motifs|PWM||Nanog, Chromatin_Structure||DNase-seq|Fibrobl, Chromatin_Structure||DNase-seq|Th2	5
chr10	rs10509906	Motifs|Footprinting|H1_ES|Sp1, Motifs|Footprinting|GM12892|RREB1, Motifs|Footprinting|Lymphoblastoid|RREB1, Motifs|Footprinting|K562|RREB1, Motifs|Footprinting|HUVEC|RREB1, Motifs|Footprinting|GM12891|RREB1	6
chr1	rs708605	Motifs|PWM||FOXP1	6

score* :The smaller the annotation number of the SNP, the more likely of the SNP to be functional regulated.

Supplementary Table 2. Detailed clinical information of the subjects in this study

	Age range （Months）	Age ≤3	Age >3	Females	Males	Cystic biliary atresia （CBA）	non-CBA
Cases (n=506)	2.088±1.934	470	36	214	292	44	462
%		92.89%	7.11%	42.29%	57.71%	8.70%	91.30%
Controls (n=1473)	18.61±19.75	521	952	44	462		
		35.37%	64.63%	8.70%	91.30%		
